# Type I vs type II spiral ganglion neurons exhibit differential survival and neuritogenesis during cochlear development

**DOI:** 10.1186/1749-8104-6-33

**Published:** 2011-10-11

**Authors:** Meagan Barclay, Allen F Ryan, Gary D Housley

**Affiliations:** 1 Department of Physiology, The University of Auckland, Private Bag 92019, Auckland, New Zealand; 2 Departments of Surgery and Neurosciences, San Diego VA Medical Center and University of California, San Diego, La Jolla, CA, 92093-0666, USA; 3 Translational Neuroscience Facility and Department of Physiology, School of Medical Sciences, University of New South Wales, Sydney, NSW 2052, Australia

## Abstract

**Background:**

The mechanisms that consolidate neural circuitry are a major focus of neuroscience. In the mammalian cochlea, the refinement of spiral ganglion neuron (SGN) innervation to the inner hair cells (by type I SGNs) and the outer hair cells (by type II SGNs) is accompanied by a 25% loss of SGNs.

**Results:**

We investigated the segregation of neuronal loss in the mouse cochlea using β-tubulin and peripherin antisera to immunolabel all SGNs and selectively type II SGNs, respectively, and discovered that it is the type II SGN population that is predominately lost within the first postnatal week. Developmental neuronal loss has been attributed to the decline in neurotrophin expression by the target hair cells during this period, so we next examined survival of SGN sub-populations using tissue culture of the mid apex-mid turn region of neonatal mouse cochleae. In organotypic culture for 48 hours from postnatal day 1, endogenous trophic support from the organ of Corti proved sufficient to maintain all type II SGNs; however, a large proportion of type I SGNs were lost. Culture of the spiral ganglion as an explant, with removal of the organ of Corti, led to loss of the majority of both SGN sub-types. Brain-derived neurotrophic factor (BDNF) added as a supplement to the media rescued a significant proportion of the SGNs, particularly the type II SGNs, which also showed increased neuritogenesis. The known decline in BDNF production by the rodent sensory epithelium after birth is therefore a likely mediator of type II neuron apoptosis.

**Conclusion:**

Our study thus indicates that BDNF supply from the organ of Corti supports consolidation of type II innervation in the neonatal mouse cochlea. In contrast, type I SGNs likely rely on additional sources for trophic support.

## Background

Development of the nervous system is characterized by the pruning of inappropriate contacts through synapse elimination [1], axon retraction [2] and by apoptosis of neurons [3,4]. In the cochlea, these processes refine afferent innervation of the sensory hair cells during early development, resulting in the precise pattern of innervation observed in the adult.

The mature mammalian cochlea exhibits segregated innervation of its two populations of sensory hair cells by the spiral ganglion neurons (SGNs), the primary auditory neurons. Type I SGNs comprise 90 to 95% of the SGN population and 10 to 20 of these neurons extend single, unbranched, myelinated neurites to exclusively innervate a single inner hair cell (IHC). The IHCs with their highly convergent and exclusively type I SGN innervation are responsible for encoding sound stimuli. The remaining 5 to 10% of SGNs are type II neurons that extend thin, unmyelinated fibers that innervate numerous outer hair cells (OHCs) in an *en passant *fashion. Auditory coding from the type II SGNs has not been determined but it is thought to signal the operating point of the 'cochlear amplifier' or active enhancement of sound transduction that is associated with the OHC [5,6]. This highly conserved afferent innervation of IHCs and OHCs is established during the first postnatal week of development in rodents. Type I arbors, which initially project to both hair cell types, withdraw from the OHC region and from adjacent IHCs to focus on a single IHC [7-11]. Type II fibers lose any contact to the IHC region, turn basally, a process that requires expression of the transcription factor *Prox1 *[12], and increase their length five-fold as they extend within the outer spiral bundles beneath the rows of OHCs [7]. This postnatal period is also known to be characterized by apoptosis of approximately 25% of SGNs [13,14].

Studies in the visual system of chicks show that programmed cell death provides a means of removing neurites that have innervated inappropriate targets [15] and is thought to arise from restriction of neurotrophin supply from target tissue [3]. In the developing inner ear, brain-derived neurotrophic factor (BDNF) and neurotrophin-3 (NT-3) are necessary for the survival of SGNs [16-18]. Both BDNF and NT-3 are expressed by the sensory hair cells in the developing organ of Corti. In the mouse, NT-3 is broadly expressed in the organ of Corti at birth (with an apically biased gradient), becoming constrained to the inner hair cells and adjacent supporting cells after the onset of hearing (second postnatal week) [19]. At birth, BDNF expression is largely constrained to the IHCs and OHCs [20] and is then down-regulated from the basal turn upwards, reflecting progressive maturation of the developing organ of Corti [21,22]. Null mutants for BDNF and NT-3, or their respective TrkB and TrkC receptors, cause a loss of SGNs during embryogenesis [23-25]; disruption of BDNF/TrkB signaling particularly affects type II SGNs and their innervation of OHCs. Knock-in of BDNF expression under the NT-3 promoter [26] and, conversely, knock-in of NT-3 under the BDNF promoter [27] indicated that BDNF, or NT-3 substituting for the BDNF spatiotemporal expression profile in the neonatal cochlea, supports both type I and type II SGN survival and the development of the type II SGN innervation of the OHC.

Our study sought to clarify the molecular signaling that determines how the two different populations of primary afferent neurons segregate in the developing cochlea to innervate their respective targets, the IHCs and the OHCs. We utilized the mouse model, which benefits from the selective expression by type II SGNs of peripherin, a type III intermediate filament [8]. By immunolabeling peripherin-positive type II SGNs and comparing this with immunolabeling of both populations of SGNs with an anti-β-tubulin antibody, we were able to determine the fate of the two SGN neuron populations *in vivo *and *in vitro *during the critical final resolution of afferent innervation of the cochlea in the first postnatal week, just prior to the onset of hearing. Our findings reveal a differential dependency on BDNF neurotrophic support for survival and neuritogenesis of the type II versus type I spiral ganglion afferent neurons.

## Results

**Figure 1:**
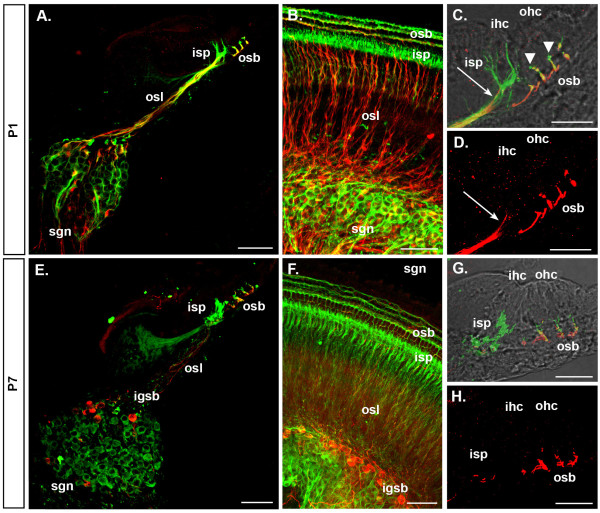
**Double labeling for β-tubulin and peripherin distinguishes type I and type II spiral ganglion neurons (SGNs) and their peripheral innervation during the first postnatal week of mouse development**. Single plane (A,C,D,E,G,H) and maximum projections (B,F) of confocal image stacks show β-tubulin (green) and peripherin (red) immunofluorescence in cochleae from P1 (A-D) and P7 (E-H) mice. **(A-D) **At P1 peripheral fibers from type I SGNs (immunolabeled for β-tubulin only) form the inner spiral plexus (isp) beneath the inner hair cell (ihc). Type II SGN soma and peripheral fibers (β-tubulin and peripherin immunolabeled) are numerous in the spiral ganglion (sgn) and osseus spiral lamina (osl) of P1 cochleae (A,B). Their neurites are present beneath the IHCs (arrow in (C,D)) and beneath the outer hair cells (ohc) in the outer spiral bundles (osb). **(E-H) **At P7, the majority of neurons in the spiral ganglion are type I neurons (E,F) and their neurites innervate the IHCs (G). Peripherin and β-tubulin double-immunolabeled type II SGNs are now localized laterally in the spiral ganglion near the intra-ganglionic spiral bundle (igsb), their fibers form the outer spiral bundle and innervate the OHC only; no peripherin-immunolabeled fibers extend toward the IHC (H). Variation in the color (yellow to red) of double labeled (type II) neuron soma and their neurite processes arises from differences in the relative intensity of the β-tubulin (green fluorescence) in soma and neurites and also peripherin (red) fluorescence in merged confocal images. This is particularly evident at P7, where the type II soma appear red as there is a decline in β-tubulin labeling, whereas the outer spiral bundle region is yellow, due to approximate equivalence of the intensity of the red and green immunofluorescence in this region (see (E)). We attribute double labeling (type II SGNs) versus single (β-tubulin) labeling (type I SGNs) from analysis of individual channels. Panels (A,C-E,G,H) are from cryosectioned tissue; (B,F) are whole-mount preparations. Scale bars: 50 μm (A,B,E,F); 25 μm (C,D,G,H).

In this study we investigated the density of type I and type II SGNs *in vivo *and *in vitro *to gain further insight into the mechanisms that maintain these neuronal sub-types during postnatal development. β-Tubulin antiserum allowed identification of the total SGN population [28-34] and double immunolabeling for peripherin distinguished the type II SGNs and their neurites [8] in cochlear tissue from postnatal day (P)1 and P7 mice compared with P21 as a mature reference. This period encompassed the refinement of afferent innervation in the mouse cochlea (Figure 1) [8].

### *In vivo *spiral ganglion neuron density

**Figure 2:**
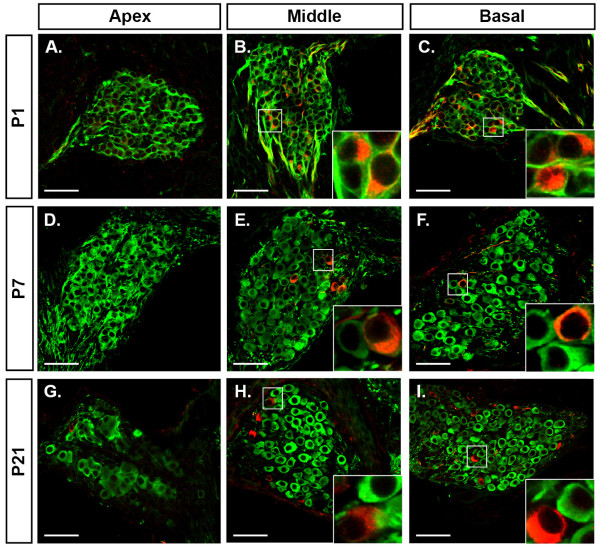
**Peripherin immunolabelled type II spiral ganglion neurons (SGNs) are absent at the apex of the cochlea and between P1 and P7 their number reduces in the middle and basal regions**. **(A-I) **Single plane confocal images of β-tubulin (green) and peripherin (red) double immunolabeling of SGNs in the most apical region (apex) (A,D,G), and in the middle (B,E,H) and basal (C,F,I) turns in sectioned cochleae from P1 (A-C), P7 (D-E) and P21 (G-I) mice. Marked areas are shown in insets and detail β-tubulin and peripherin immunolabeling of SGNs. β-Tubulin and peripherin double immunolabeled type II SGNs are absent from the apex of P1 (A), P7 (D) and P21 (G) mouse cochleae. Many of these double-immunolabeled type II SGNs are present in the middle and basal turns in P1 cochlea (B,C); however, this number is reduced in P7 (E,F) and P21 (H,I) cochleae. The level of β-tubulin expression in the type II SGN soma appears reduced in P7 and P21 cochleae, resulting in relatively stronger red signal (peripherin channel). Scale bars: 50 μm.

At P1 the spiral ganglion contained many type II SGNs, identified as β-tubulin and peripherin double-immunolabeled (Figures 1A,B and 2A-C). The type II SGNs had somata distributed throughout the ganglion and neurites predominantly projecting to OHCs, with minor side projections to IHCs (Figure 1C,D). The type I SGN somata were the majority, and their fibers (immunolabeled for β-tubulin but not peripherin) formed a dense inner spiral plexus (ISP) at the base of the IHCs. In contrast, cochleae from P7 mice exhibited a reduced number of peripherin immunolabeled type II SGNs (Figure 2E,F). These type II SGNs were localized near the intraganglionic spiral bundle and β-tubulin expression in their soma appeared reduced in P7 (Figures 1E,F and 2E,F) and P21 (Figure 2H,I) cochleae and reflects previous findings by Lallemend and colleagues [31], who showed a downregulation of this protein in rat type II SGNs from P5. Type II fibers innervated only the OHCs (Figure 1E-H), matching the adult-like distribution at P21 (Figure 2G-I). There was no evidence of the type II fiber innervation of the IHCs that was observed at P1 (arrow in Figure 1C,D). Type I SGN innervation was consolidated at the ISP, associated with the basolateral region of the IHC (Figure 1E-G).

**Figure 3:**
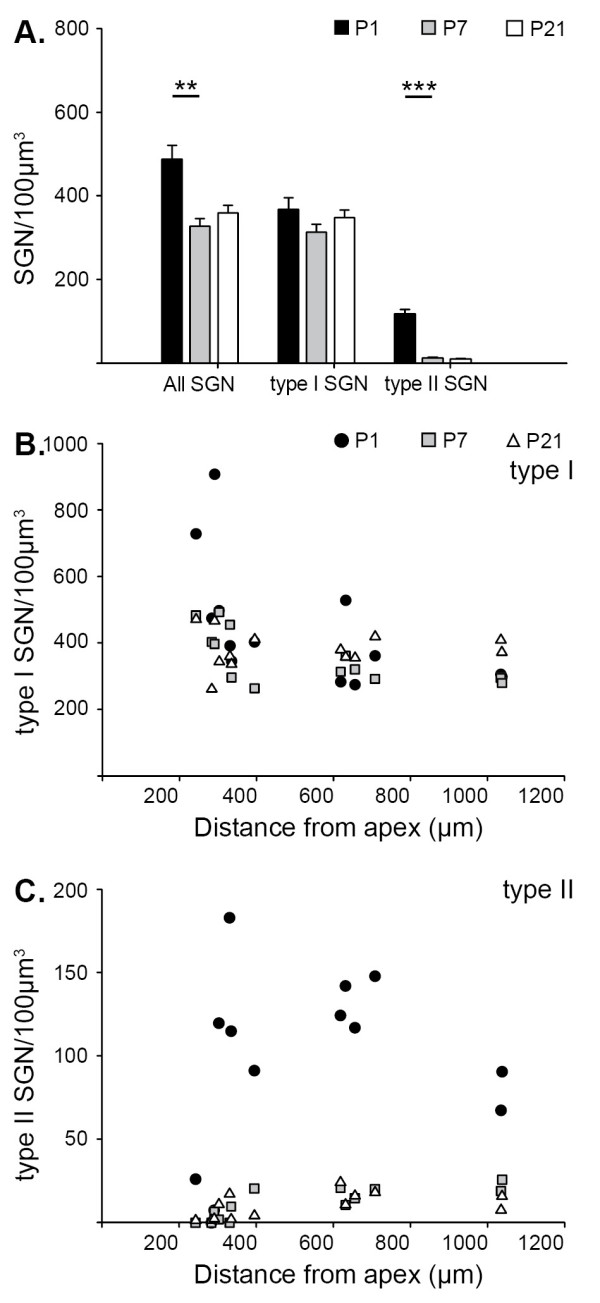
**Quantitative analysis of type I and type II spiral ganglion neuron (SGN) densities in P1, P7 and P21 mouse cochlea shows selective loss of type II SGNs in the first postnatal week *in vivo***. **(A) **Density of all SGNs, type I SGNs and type II SGNs calculated from cell counts taken from mid-modiolar cross-sections from P1 (black), P7 (grey) and P21 (white) mouse cochlea. Mean density ± standard error of the mean; ANOVA, *post hoc *Holm-Sidak test, **P < 0.01, ***P < 0.001. **(B,C) **The density of type I (B) and type II (C) SGNs within different regions of the spiral ganglion in mid-modiolar cross-sections are plotted against the distance of the ganglia from the apex of the cochlea.

To further investigate the apparent loss of peripherin immunolabeled type II SGNs during postnatal development, we examined the density of type I and type II SGNs in spiral ganglia from cryosectioned P1 (n = 4), P7 (n = 5) and P21 (n = 5) mouse cochleae (Figures 2 and 3). The most apical region of the spiral ganglia lacked peripherin-positive type II SGNs in P1 (Figures 2A and 3C), P7 (Figures 2D and 3C) and P21 (Figures 2G and 3C) mice.

β-Tubulin expression permitted quantification of neuron density for the total SGN population (Figures 2 and 3A), which showed an approximately 25% reduction in density between P1 and P21 (P1 = 497 ± 51 neurons/100 μm^3^; P7 = 327 ± 11 neurons/100 μm^3^; P21 = 363 ± 28 neurons/100 μm^3^; one-way ANOVA, *P *< 0.01). This significant loss of SGNs occurred between P1 and P7 (*post-hoc *Holm-Sidak test, *P *< 0.05). Type I SGNs, which expressed β-tubulin only (Figure 2), showed neuron densities of 376 ± 92 neurons/100 μm^3^, 312 ± 27 neurons/100 μm^3 ^and 352 ± 28 neurons/100 μm^3 ^for P1, P7 and P21 cochleae, respectively (Figure 3A), and were not significantly different (one-way ANOVA, *P *= 0.268). Peripherin and β-tubulin immuno-positive type II SGNs (Figure 2), however, showed a marked and significant reduction in neuron density between P1 and P21 (Figure 3A; one-way ANOVA, *P *< 0.001). *Post hoc *analyses revealed this significant loss of type II SGNs occurred between P1 and P7, where their density dropped from 121 ± 21 neurons/100 μm^3 ^to 15 ± 3 neurons/100 μm^3 ^(Holm-Sidak test, *P *< 0.05; 88% loss). No further reduction was observed at P21, when type II SGN density was 15 ± 4 neurons/100 μm^3^. Thus, the type II SGN population reduces from approximately 26% of all SGNs at P1 to approximately 5% at P7. There was no marked tonotopic effect on neuron density when the density of type I and type II SGNs in each ganglion region was plotted with respect to the distance from the apex (Figure 3B and Figure 3C, respectively). These plots confirm that type I SGNs show very little change in neuron density postnatally (Figure 3B), but the five-fold reduction in the number of type II SGNs noted above occurs within the first postnatal week, across all turns of the cochlea (excluding the very apex, where there are no type II SGNs even at P1) (Figure 3C).

### Spiral ganglion survival *in vitro*

**Figure 4:**
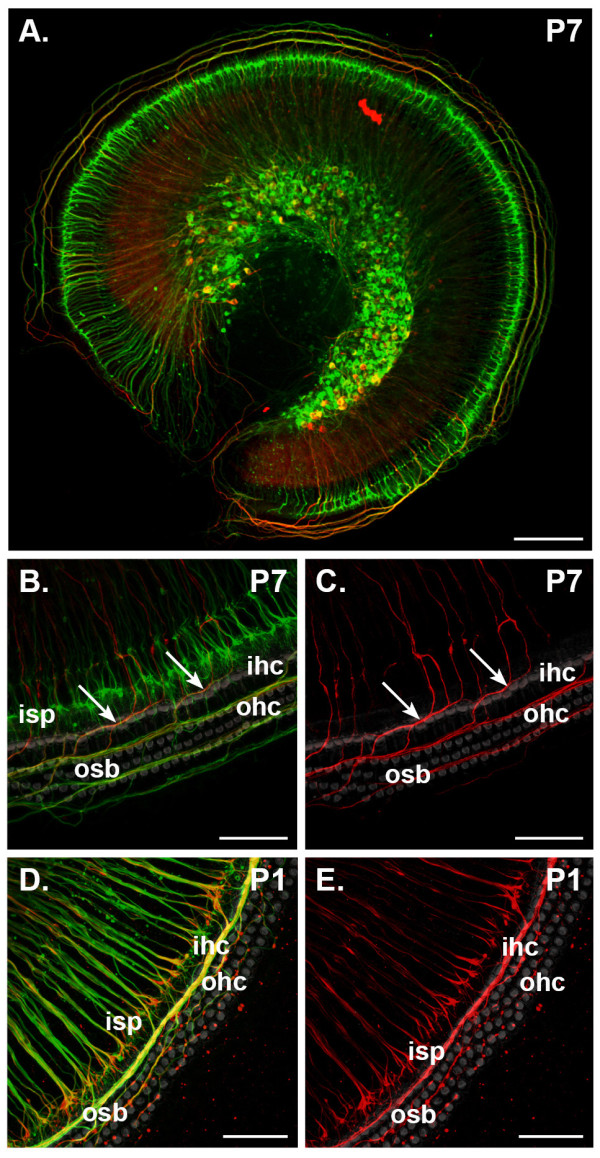
**The expression pattern of β-tubulin and peripherin in type I and type II spiral ganglion neurons is maintained *in vitro***. Maximum intensity projections from confocal z-stacks show immunofluorescence labeling for β-tubulin (green) and peripherin (red) in the mid-apex-mid-turn region of P1 (D,E) and P7 (A-C) mouse cochlea following 48 hour organotypic culture (SGNs and organ of Corti intact). Rhodamine-phalloidin labeling (grey in (B-E)) confirmed the survival of target hair cells. **(A,B) **Immunolabeling of organotypic cultures of P7 tissue shows the inner spiral plexus (isp), formed by type I fibers, contains β-tubulin protein only (detailed in (B)), whereas the outer spiral bundles (osb) that arise from type II fibers express both β-tubulin and peripherin (arrows in (B,C)). **(C) **Examination of peripherin immunofluorescence alone confirms that this protein is expressed only in fibers that cross the tunnel of Corti and innervate the outer hair cells (ohc). **(D,E) **Confocal imaging of organotypic culture of P1 cochlear tissue shows that singularly β-tubulin immunofluorescent fibers beneath the inner hair cells (ihc) are reduced in density compared to the *in vivo *situation (compare (D) with Figure 1B). A large portion of these fibers co-immunolabel for peripherin (D,E). The outer spiral bundle double immunolabels with β-tubulin and peripherin (D,E). Scale bars: 150 μm (A); 50 μm (C-E).

Apoptosis is often controlled by the supply of trophic support to afferent fibers by target tissue following synaptogenesis [35]. To examine how peripheral trophic support from the hair cells of the organ of Corti might maintain type I and type II SGNs during the first postnatal week of development, we investigated the survival of these SGN sub-types in organotypic and explant cultures where the organ of Corti was intact, or removed, respectively. Subsequently the trophic effect of exogenous BDNF on SGN subtypes in explant cultures was examined. We first examined β-tubulin and peripherin immunoreactivity in SGNs and their peripheral neurites to confirm that peripherin expression remained specific to mouse type II SGNs following 48 hours of culture (Figure 4). Organotypic cultures of P7 cochleae - where, *in vivo*, type I fibers innervate the IHC and type II fibers innervate just the OHC - showed that the associated differential immunolabeling was retained. Thus, in P7 organotypic tissue culture preparations, the IHC innervation (ISP region) exhibited only β-tubulin immunolabeling - consistent with type I SGN fibers (Figure 4A,B) - whereas double immunolabeling for peripherin and β-tubulin (of type II fibers) was similarly constrained to the region of OHC innervation (the outer spiral bundle) (Figure 4B,C).

**Figure 5:**
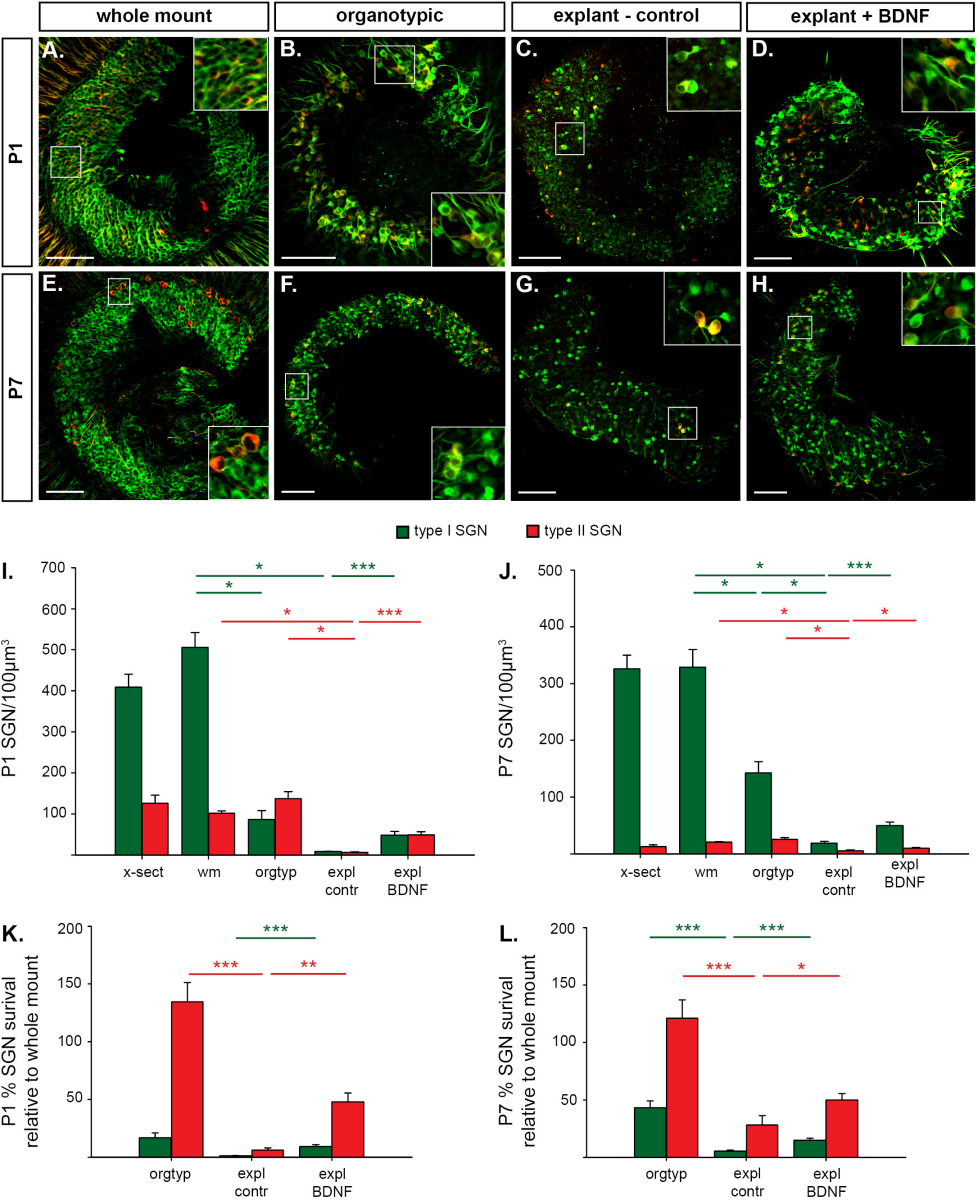
**The organ of Corti (oC) maintains type II spiral ganglion neurons (SGNs), but not type I SGNs in organotypic culture and brain-derived neurotrophic factor (BDNF) rescues a greater proportion of type II SGNs following explant culture without the oC**. **(A- H) **Single plane confocal images of β-tubulin (green) and peripherin (red) immunofluorescence in the mid-apex-mid-turn region of cochleae from P1 (A-D) and P7 (E-H) mice enabled analysis of type I SGN (immunolabel for β-tubulin only) and type II SGN (immunolabel for β-tubulin and peripherin) density. SGN density in the mid-apex-mid-turn region *in vivo *was determined in whole mount tissue (whole mount) (A,E). Survival of SGN sub-types was subsequently established following 48 hour culture of cochlear tissue: retaining both SGNs and oC (organotypic) (B,F); just the spiral ganglion, following removal of the oC (explant - control) (C,G) and this spiral ganglion explant cultured with 100 ng/ml BDNF (explant + BDNF) (D,H). Inset shows marked area in corresponding panel at higher magnification and shows detail of type I and type II SGNs. **(I,J) **Neuron densities for type I (green) and type II (red) SGNs from P1 (I) and P7 (J) mice are plotted for each condition shown in panels (A-H). x-sect, mid-apex-mid-turn ganglion in mid-modiolar cross-sections; wm, whole mount; orgtyp, organotypic; expl BDNF, explant + BDNF; expl contr, explant - control. Mean density and standard error of the mean; one-way ANOVA with *post hoc *Holm Sidak tests compare SGN survival in the wm, orgtyp and expl contr; unpaired *t*-tests compare expl contr and expl BDNF; *P < 0.05, ***P < 0.001. **(K,L) **Percentage survival of type I and type II SGNs in orgtyp, expl BDNF and expl contr relative to mean *in vivo *whole mount densities are plotted for P1 (K) and P7 (L) mice. Mean density and standard error of the mean; unpaired *t*-test, *P < 0.05, **P < 0.01, ***P < 0.001. Scale bars: 250 μm.

Organotypic culture of P1 mouse cochlear tissue gave rise to re-organization of the outer spiral bundle fibers, as there was dense type II fiber labeling (β-tubulin and peripherin) near the first row of OHCs (Figure 4D). The ISP was composed of fibers that expressed either β-tubulin only, or both peripherin and β-tubulin (Figure 4D). β-Tubulin and peripherin double-immunolabeled fibers in the ISP (Figure 4D) appeared more prominent than what we observed in *in vivo *P1 organ of Corti (Figure 1B) and β-tubulin-only-expressing type I fibers in the ISP were diminished in density (Figure 4E) relative to what we observed *in vivo *(Figure 1). This likely reflected the significant loss of type I SGNs following organotypic culture (Figure 5).

To investigate the pro-survival effect of the organ of Corti on type I and type II SGNs, we assessed the *in vivo *density of these neuronal sub-types in whole mounts of the mid-apex-mid-turn region of cochleae from P1 (Figure 5A) and P7 (Figure 5E) mice. These densities were compared to those determined for the spiral ganglion following 48 hour culture in control media with the organ of Corti intact (organotypic, Figure 5B,F), or removed (explant - control, Figure 5C,G). The effect of BDNF supplement in the culture media on type I and type II SGN survival was also investigated in SGN explants (explant plus BDNF, Figure 5D,H).

We validated our methods for calculating neuron density by comparing the *in vivo *density of type I and type II SGNs in the mid-apex-mid-turn region across two different fresh-fixed tissue preparations; mid-modiolar cross-sections (P1, n = 4; P7, n = 5) and whole mount tissue (P1, n = 5; P7, n = 6), where we found no significant difference in neuron density for each SGN sub-type (Figure 5I,J). Forty-eight hour culture of type I SGNs from P1 cochleae with the organ of Corti intact (organotypic, n = 4) or removed (explant, n = 7) yielded significant decreases in neuron density when compared to *in vivo *densities calculated from whole mount tissue (Figure 5I; whole mount tissue = 506 ± 36 neurons/100 μm^3^; organotypic = 87 ± 22 neurons/100 μm^3^; explant = 9 ± 1 neurons/100 μm^3^; ANOVA, *P *< 0.001). This loss of neurons arose in both organotypic and explant cultures (*P *< 0.05, *post hoc *Holm Sidak test). The application of BDNF to SGN explants (n = 7) improved survival of cultured type I SGNs to 49 ± 9 neurons/100 μm^3 ^when compared to the control explants (Figure 5I; unpaired *t*-test, *P *< 0.001). In contrast, at P1, while type II SGNs also showed a significant effect of culture on neuron density (Figure 5I; whole mount tissue = 103 ± 6 neurons/100 μm^3^; organotypic = 138 ± 34 neurons/100 μm^3^; explant = 7 ± 2 neurons/100 μm^3^; ANOVA, *P *< 0.001), this loss of neuron viability only occurred in the explant condition (*P *< 0.05, *post hoc *Holm Sidak test). Thus, retention of the organ of Corti in the organotypic culture preparations provided sufficient support for complete survival of the type II SGN population. The loss of type II SGNs in the explant preparation was rescued by BDNF (Figure 5I; density was increased to 50 ± 8 neurons/100 μm^3^; unpaired *t*-test, *P *< 0.001), or about 50% survival, a considerably higher proportion than the BDNF-mediated rescue of type I SGNs in explants (Figure 5K).

Similar patterns of survival were observed in whole mount (n = 4), organotypic (n = 7) and explant (n = 5) tissue from P7 mice (Figure 5J). Type I SGNs again showed a significant reduction in neuron density with culture (whole mount tissue = 329 ± 32 neurons/100 μm^3^; organotypic = 143 ± 20 neurons/100 μm^3^; explant = 20 ± 3 neurons/100 μm^3^; ANOVA, *p *< 0.001) that arose in both organotypic and explant cultures (*P *< 0.05, *post hoc *Holm Sidak tests). BDNF yielded a significant improvement in type I SGN survival, where an increase of neuron density to 51 ± 6 type I neurons/100 μm^3 ^was observed when compared to control explants (Figure 5J; unpaired *t*-test, *P *< 0.01). Type II SGNs also showed a significant effect of culture on neuron density (Figure 5J; whole mount tissue = 22 ± 1 neurons/100 μm^3^; organotypic = 26 ± 3 neurons/100 μm^3^; explant = 6 ± 2 neurons/100 μm^3^; ANOVA, *P *< 0.001). Again it was the explant cultures that exhibited a significant loss of neurons when compared to the *in vivo *neuron density (whole mount tissue; *P *< 0.05, *post hoc *Holm Sidak test) and culture of type II SGNs with the organ of Corti intact resulted in no significant change in their density, indicating complete protection from type II SGN loss. Culture of P7 SGN explants (no organ of Corti) with BDNF significantly improved type II SGN survival when compared to P7 explant cultures in control growth media (Figure 5J; 11 ± 1 type II neurons/100 μm^3^; unpaired *t*-test, *P *< 0.05). As for experiments with P1 tissue, these P7 experiments again showed that proportionally more type II neurons were rescued by BDNF than type I SGNs (Figure 5L). Further, the complete survival of type II SGNs in the P1 and P7 organotypic cultures validated our *in vitro *neuronal density quantification.

### Spiral ganglion neuron neuritogenesis *in vitro*

**Figure 6:**
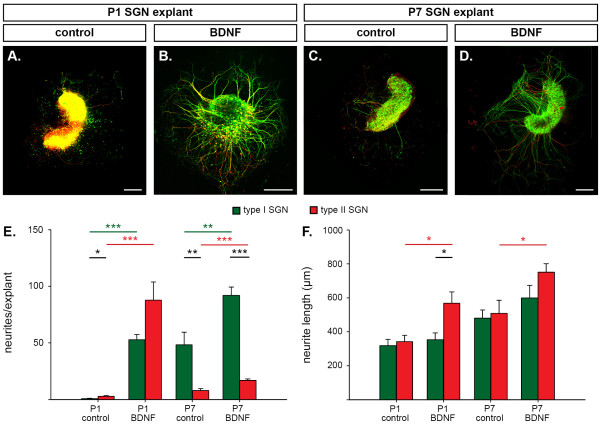
**Brain-derived neurotrophic factor (BDNF) promotes neuritogenesis of both type I and type II spiral ganglion neurons (SGNs) in explants, but has a greater effect on type II SGNs**. **(A-D) **Analysis of neurite outgrowth in mid-apical-mid-turn region explants from P1 (A,B) and P7 (C,D) mouse cochlea following 48 hour culture in control culture media (A,C) or with 100 ng/ml BDNF added to the media (B,D). Confocal immunofluorescence images show type I SGN neurites (β-tubulin (green) only) and type II SGN neurites (β-tubulin and peripherin (red)). **(E,F) **BDNF stimulated an increased number of type I (green) and type II (red) neurites per explant (E) and significantly longer type II neurites (F). The neurites extend from the explants in bundles and then separate into individual fibers. This gives the appearance of branching towards the ends in the low magnification images. Data are represented as mean and standard error of the mean; unpaired *t*-test, *P < 0.05, **P < 0.01, ***P < 0.001. Scale bars: 250 μm.

Neurite outgrowth from spiral ganglia explants cultured in control growth media (P1, n = 7; P7, n = 11) was compared with that in explants with BDNF-supplemented culture medium (P1, n = 7; P7, n = 10). Minimal neurite outgrowth was observed when P1 and P7 explants were cultured in control media (Figure 6A and Figure 6C, respectively); however, the addition of BDNF led to extensive neurite outgrowth at both P1 and P7 (Figure 6B and Figure 6D, respectively). The numbers of neurites extending from each explant were counted and the mean values ± standard error of the mean across explants showed that the culture of the P1 mouse cochlea with BDNF yielded highly significant increases in the number of both type I and type II neurites (Figure 6E). Type I neurites increased from 1 ± 0 to 53 ± 5 neurites/explant (unpaired *t*-test, *P *< 0.001) and type II neurites increased from 3 ± 1 to 88 ± 16 neurites/explant. P7 explants showed a similar, although relatively more modest, effect (Figure 6E); the number of type I neurites increased from 49 ± 11 neurites/explant to 92 ± 7 neurites/explant with the addition of BDNF to the growth media (unpaired *t*-test, *P *< 0.01) and type II neurites increased significantly from 8 ± 2 neurites/explant to 17 ± 1 neurites/explant (unpaired *t*-test, *P *< 0.001). There were significantly more type I neurites than type II neurites in both control (unpaired *t*-test, *P *< 0.05) and BDNF-treated (unpaired *t*-test, *P *< 0.001) explants from P7 cochleae, which reflects the reduction in numbers of type II SGNs in the explants at this later age.

To investigate the effect of BDNF on the length of type I and type II neurites, mean neurite length for these SGN sub-types was calculated for each explant (Figure 6F). Comparison of the mean lengths of type I and type II neurites grown in control growth media were not significantly different, regardless of age. Type I and type II SGN neurites from P1 mice had a mean length of 320 ± 38 μm and 343 ± 38 μm, respectively, and those from P7 mice had a mean length of 481 ± 48 μm and 510 ± 77 μm, respectively. Addition of BDNF to the culture medium stimulated a significant increase in type II neurite length to 569 ± 67 μm in P1 explants (unpaired *t*-test, *P *< 0.05) and to 752 ± 50 μm in P7 explants (unpaired *t*-test, *P *< 0.05). Type I neurites showed no BDNF-mediated increase in length (354 ± 40 μm at P1 and 600 ± 74 μm at P7; *P *> 0.05).

## Discussion

Neonatal mouse cochlea provide a unique opportunity in which to distinguish type I and type II SGNs using peripherin as a specific marker for type II SGNs. We show that approximately 25% of neurons at birth are peripherin-positive and therefore type II SGNs. The apical-most half turn of the mouse cochlea did not contain peripherin-immunolabeled type II SGNs, in agreement with previous studies [36,37]. Our results also agree with the previous study by Huang *et al. *[8] that first demonstrated the specificity of peripherin immunolabeling in the mouse cochlear type II SGN from embryonic day 18. As shown by Huang *et al*., the peripherin-positive (type II SGN) neurites, which project beyond the floor of the tunnel of Corti to the OHCs, formed distinctly separate fascicles from the transient innervation of the OHCs by the peripherin-negative type I SGNs in the first postnatal week. This reflects a species difference, as in the neonatal rat model, peripherin expression occurs in type I as well as type II SGNs [36,31]. We are also confident that peripherin expression remained confined to the type II SGNs following axotomy and culture, despite previous reports of *in vitro *peripherin re-expression in type I SGNs from rat cochlea following axotomy and culture of SGN explants [31] and *in vivo *following degeneration of the organ of Corti in response to neomycin treatment [38]. In particular, we did not observe peripherin expression in fibers innervating IHCs in our organotypic cultures from P7 cochleae, by which age the IHC innervation is exclusively from type I SGNs [7]. Additionally, the density of peripherin-expressing type II SGNs in these cultures matched the *in vivo *state, again suggesting that type I SGNs in our cultures did not start to express peripherin. It is highly unlikely that exactly sufficient numbers of type I SGN could start to express peripherin, to balance putative loss of type II SGN, so that the peripherin-expressing neuron population remained unchanged. It is also highly unlikely that such a process would exactly match different proportional loss of type II SGNs at P1 versus P7. These findings are corroborated by previous studies in neonatal mouse cultures, where peripherin was never observed in type I fibers innervating the IHCs in organotypic cultures [39] and where, following electrophysiological recording from 228 SGNs in explant cultures, only 9 neurons were unequivocally identified as type II SGNs based on their peripherin expression [32]. The peripherin-expressing fibers that we observed in the ISP region at P1 likely represent type II fiber arbors that were noted following organotypic culture of postnatal mouse cochlear tissue [40].

### Selective loss of type II SGNs during the first postnatal week of development

Our data indicate that most of the known loss of approximately 25% of SGNs *in vivo *that occurs during the first postnatal week [13,14] is accounted for by loss of type II SGNs, and there is no significant change in type I SGN density. Previous qualitative observations indicated that at least a portion of SGN loss was accounted for by type II neurons, as identified by their peripherin expression [8] or location in Rosenthal's canal [41]. Furthermore, quantification of SGN loss in the human cochlea during early development suggested that the proportion of type II neurons decreased from 25% during early embryogenesis to 12% in the infant cochlea, based on reduction of the proportion of smaller sized SGNs that were more likely to be type II SGNs [42].

### Dependence of type I and type II SGNs on endogenous trophic support

Neuronal loss occurs extensively in the developing nervous system and is thought to provide a means of matching the size of a neuronal population with that of their postsynaptic targets through competition for limiting amounts of trophic support supplied by target tissue [3,43,44]. Studies in the cochlea have shown that neonatal SGNs rely on connectivity with their peripheral targets for survival *in vitro *[45], and recently Brn3c3.1 null mutants, which develop immature hair cells that die by apoptosis during the neonatal period [46,47], concomitantly lost more than 50% of their SGNs, including the type II SGN innervation to OHCs by P7 [48]. Our results indicate that connectivity between SGNs and the hair cells of the organ of Corti promotes the survival of both type I and type II SGNs postnatally. However, whereas trophic support supplied by the sensory epithelium was sufficient to maintain the entire population of type II SGNs, it did not support the majority of type I SGNs. This significant reduction in type I SGN density *in vitro *that we quantified may arise from differential sensitivity to disruption of SGN connectivity with the cochlear nucleus. A recent study showed that loss of neurons in especially the ventral cochlear nucleus (mediated by the conditional deletion of the *Atoh1 *gene in this region) led to the loss of approximately 30% of SGNs between P0 and P3 [49]. Although the identity of the lost SGNs was not known, substantial losses occurred in regions of the spiral ganglion that are preferentially populated by type I SGNs. Interestingly, this loss of SGN coincides with the period of initiation of BDNF and NT-3 expression in the cochlear nucleus of gerbils and rats [50,51] and the down-regulation of these neurotrophins in the organ of Corti [19,21,22], suggesting a switch of survival support from the sensory epithelium to the central target. Our data suggest that only the type I SGNs may be dependent upon the cochlear nucleus during this period. This difference in trophic dependence would help to explain the preferential loss of type II SGNs that occurs *in vivo *during this period. While the type I SGNs may be able to survive a reduction in neurotrophin production in the organ of Corti by switching dependence to a cochlear nucleus source, type II SGNs appear to be independent of their central targets and thus to be more dependent upon the declining peripheral neurotrophic support. Our finding that exogenous BDNF support provided significantly greater survival of type II SGNs than type I SGNs *in vitro *in explants at P1 corroborates the idea that the early postnatal downregulation of this neurotrophin in hair cells may give rise to the loss of type II SGNs *in vivo *[22].

### Refinement of type I and type II SGN innervation of the developing organ of Corti

In addition to changes in neuronal survival, the first postnatal week is a period of significant remodeling of SGN neurites within the organ of Corti. Type I SGN neurites retract from the region of the OHCs and focus on a single IHC, while type II SGN neurites withdraw from the IHC region and substantially lengthen their projection along the OHC region [7-11]. The reduction of projections could be mediated by either the loss of neurons or remodeling of existing neurites. Our observation that only type II SGNs are lost from the neonatal SGN *in vivo *provides clarification, as the withdrawal of type I neurites from beneath the OHCs during this period cannot be the result of neuronal loss, but must represent the retraction of collateral neurites. In contrast, the reduction in type II dendrite presence underneath the IHCs could represent the loss of type II neurons, dendrite remodeling, or both. As the type II neurites increase their projections beneath the OHCs, they have been reported to retract collateral branches extending toward the IHCs [8,52]. The specific marker of type II SGNs - the type III intermediate filament peripherin - may itself also bias this differential neurite outgrowth [28]. It is also possible that some type II neurons originally projecting to the IHCs are lost. Our data cannot distinguish between these alternatives.

Whereas BDNF supported the survival of both type I and type II SGNs, this neurotrophin exerted a significant effect only on neurite outgrowth in type II SGNs. This was particularly evident at P1, corresponding to the time when type II fibers extend into the OHC region [7,8]. The differential effect of BDNF on neuritogenesis in the two SGN populations likely reflects the interaction of downstream signaling pathways, as TrkB receptors are expressed by both type I and type II fibers [53]. The nature of these differences is not clear. However, ATP signaling through ionotropic P2X receptors, assembled as heteromers of P2X_2 _and P2X_3 _subunits, has been shown to inhibit BDNF-dependent neurite outgrowth from SGNs [54]. *In vivo *studies with transgenic mice provide compelling evidence that a primary role of BDNF is to support the type II neurons and OHC innervation throughout the cochlea as *BDNF *and *TrkB *null mice exhibit selective loss (8 to 10%) of SGNs (type II) and their fibers beneath the OHCs [23,27,55]. These studies, and the finding that substitution of NT-3 expression for BDNF under the BDNF promoter rescues the BDNF-dependent type II SGN innervation of OHCs, indicates that there is redundancy in TrkB and TrkC receptor signaling in the (type II) SGNs that respond to the spatiotemporal regulation of neurotrophins (intrinsically BDNF) [27]. This is consistent with rescue of type II SGNs by exogenous BDNF in our explant model, but does not exclude complementary support from NT-3. NT-3 has been shown to provide additive support to BDNF in promoting survival of cultured gerbil and murine type II SGNs, and is synergistic with BDNF for type I SGN survival just after birth [39]. Broader expression of BDNF, under the NT-3 promoter (knock-in) rescues the NT-3-dependent loss of SGNs in the NT-3 null mouse [22], again consistent with redundancy in Trk receptor signaling associated with the spatiotemporal compartmentalization of secretion of the neurotrophins by the hair cells and supporting cells. While the putative action of NT-3 in support of type I and type II SGNs was not examined in the present study, it is apparent from these transgenic models, and from our *in vitro *data, that BDNF signaling is central to the development of the type II innervation of OHCs.

## Conclusion

Here we provide a comprehensive description of changes in cochlear type I and type II SGNs of mouse cochlea *in vivo *and *in vitro *during the critical period for consolidation of their innervation of IHCs and OHCs, respectively, in the first postnatal week. Our data show that the substantial neuronal loss that occurs during this period is attributable to type II SGNs alone and may also contribute to the loss of their neurites from beneath the IHCs. BDNF expression in the hair cells likely contributes a large portion of trophic support that is necessary for maintenance of this (type II) SGN sub-population and may promote the extension of the outer spiral bundle fibers along the rows of OHCs just after birth. Our model of distinguishing type I and type II SGNs will allow further investigation of the differential effect of neurotrophins and guidance molecules on these SGN sub-populations. This new knowledge of the properties of the two populations of primary auditory neurons in the cochlea will bolster research on treatments for auditory neuropathy, including research on afferent regeneration.

## Materials and methods

### Animals

All procedures in the study were approved by the University of Auckland Animal Ethics Committee or by the University of New South Wales Animal Care and Ethics Committee. Cochlear tissue was obtained from P1 to P21 C57/BL6 mice. P1 to P7 animals were killed by decapitation, and older animals were killed by intraperitoneal injection of sodium pentobarbitol (90 mg/kg; Nembutal, Virbac Laboratories, East Tamaki, New Zealand).

### Fixed tissue preparation

The innervation of the cochlea *in vivo *was determined at P1, P7 and P21 by transcardial perfusion of 4% paraformaldehyde in 0.1 M phosphate buffer following flushing with normal saline containing nitroprusside (0.9% NaCl; 0.5% Na_2_[Fe(CN)_5_NO], pH 7.4). Cochleae were subsequently perfused via the oval window with 4% paraformaldehyde in 0.1 M phosphate buffer and post-fixed at 4°C overnight. Decalcification in 8% EDTA in 0.1 M phosphate buffer (pH 7.4) at room temperature was carried out for two (P7 to 14 tissue) or 3 days (P21 tissue). The apical-most half turn of the cochlear tissue was discarded and whole mount preparations constituted one full turn of the cochlea, from the mid-apex to mid-turn region. Bony capsule, lateral wall, Reissner's membrane and the modiolus were removed, leaving the intact spiral ganglion and organ of Corti. For mid-modiolar cross-sections, fixed cochleae were placed in 10% sucrose in 0.1 M phosphate-buffered saline (PBS) for 4 hours, 30% sucrose in 0.1 M PBS for 2 days and then a 1:1 mix of 30% sucrose and Tissue-Tek OCT (Miles, Diagnostics Division, Elkhart, IN, USA) for 1 hour. They were subsequently mounted in OCT, snap frozen on dry ice and cryosections were cut at 50 μm into 0.1 M PBS. Floating sections and whole mount tissue were subsequently processed for immunofluorescence.

### Organotypic and explant cultures

The survival and neurite outgrowth from neonatal (P1 to P7) SGNs were compared with and without the organ of Corti to provide trophic support, as 'organotypic' (spiral ganglion with organ of Corti intact) and 'explant' (spiral ganglion only) cultures. The cochlear otic capsule was removed and the tissue a half turn back from the apex, lacking type II SGNs, was discarded. The modiolus was cut a further full turn down to provide a mid-apical to mid-turn region. For organotypic cultures, the intact spiral ganglion, spiral limbus and organ of Corti were retained. For explant cultures, the organ of Corti and spiral limbus were removed to isolate the spiral ganglion. Spiral ganglion plus organ of Corti organotypic or SGN-only explant tissues were transferred onto glass coverslips that were pre-coated with 50 μg/ml poly-D-lysine (BD Biosciences, San Diego, CA, USA) overnight at 4°C. These were placed in single wells of a 24-well plate, each of which contained 200 μl neuron maintenance medium (DMEM (Gibco, Carlsbad, CA, USA), 10% fetal bovine serum (Gibco), 10 μl/ml N2 supplement (Invitrogen, Carlsbad, CA, USA), 25 mM Hepes (Sigma-Aldrich, St Louis, MO, USA), 300 units/ml penicillin (Sigma-Aldrich), 6 mg/ml glucose (Sigma-Aldrich)). Medium was replaced after the first 24 hours. In explant experiments, half of the explants also contained 100 ng/ml of the neurotrophin BDNF (Promokine, Heidelberg, Germany), which was added to the cell culture medium just prior to plating. Explants were cultured at 37°C in a humidified incubator with 5% CO_2 _for 48 hours. Tissue was then fixed for 20 minutes with 4% paraformaldehyde, washed with PBS and processed for immunofluorescence.

### Immunofluorescence

Whole mounts, cryosections and cultured tissue were incubated in blocking/permeablizing solution (10% normal goat serum and 2.5% Triton X-100 in PBS) for 2 to 3 hours at room temperature and then overnight at room temperature in primary antibody solution: β-tubulin antisera (1:500 dilution) and peripherin antisera (1:1,000 dilution) with 5% normal goat serum and 0.25% Triton X-100 in 0.1 M PBS. The following day, sections were washed in PBS, then incubated for 2 hours at room temperature in secondary antibody solution (Alexa-488 conjugated goat anti-mouse IgG (1:500); Alexa-594 conjugated goat anti-rabbit IgG (1:500); Alexa-647 conjugated goat anti-rabbit (1:200); all from Invitrogen) with 5% normal goat serum and 0.25% Triton X-100 in 0.1 M PBS. Cochlear tissue was washed with PBS and in some experiments hair cells were stained with 1:150 rhodamine-phalloidin (Invitrogen) in PBS for 15 minutes at room temperature. Following further washing with PBS, tissue was mounted on glass slides in Vectastain (Vector Laboratories, Burlingane, CA, USA) and stored at 4°C.

### Background data on primary antibodies

A mouse monoclonal antibody against class III β-tubulin (TUJ1 clone, Covance, Emeryville, CA, USA) was used to label all SGNs and the expression we observed reflected that previously described for rodent cochlear tissue *in vivo *and *in vitro *[29,31]. Peripherin polyclonal rabbit antiserum (PII/SE411; a gift from Dr Annie Wolff, Division de Biochimie, Universite Pierre et Marie Curie, Paris, France) was used to distinguish the type II SGNs. This antibody specifically targets a peptide corresponding to residues 432 to 461 of rat peripherin and our immunolabeling of *in vivo *peripherin expression by the type II SGNs matched that described by Huang *et al. *[8], where the same peripherin antiserum was used. Our peripherin immunoreactivity was also equivalent to that previously described in cultured mouse cochlear tissue [39]. We have also previously validated the specificity of the peripherin antibody using peripherin null mice [56].

### Image acquisition and quantitative analysis

Images were acquired by confocal microscopy (Olympus FV1000, Japan and Zeiss LSM710, Germany), saved as TIFF files and processed using Adobe Photoshop CS2 (Adobe Systems, San Jose, CA, USA) and Image J (NIH, USA) software. Levels were adjusted to remove background fluorescence, whilst ensuring that no neurite detail was lost. Image J was used for all quantitative analysis.

*In vivo *type I and type II SGN density was quantified from mid-modiolar cross-sections of cochleae from P1, P7 and P21 mice. The apex, apical, middle and basal turn spiral ganglia in 50 μm cross-sections were imaged using confocal microscopy with 5 μm between each optical section. The volume of each ganglion was calculated by measuring the area of the spiral ganglion in each optical section, converting this to a volume by multiplying by 5 μm and summing all of the volumes within an image stack. β-Tubulin and peripherin provided good perinuclear immunolabeling of neurons and thus permitted distinction of the nuclei in each cell. Cells were counted in an optical section where the area of the nucleus was greatest relative to the soma. For example, analysis of nucleus area:soma area in consecutive optical sections revealed that 5 μm z steps were sufficient to ensure that in all cases the large round nucleus (mean relative nucleus area at maximum size = 51%) could only appear in a single image; sections above or below that showed only cytoplasm, or a small nucleus that made up less than 34% of the cell area (n = 50); thus, we were confident we were not counting a cell more than once. Neuron counts from each optical section were summed for each image stack to give a neuron count for each ganglion. Neuron density (per 100 μm^3^) was subsequently calculated. Our volumetric approach encompassed variation in SGN somata size and increases in the size of the spiral ganglion with location and age, previously observed in the gerbil cochlea between P0 and P21 [14]. The mean neuron densities of all SGNs, and separately type I SGNs and type II SGNs, were determined for each cochlea and mean neuron density for each age was subsequently calculated. Neuron densities in whole mounts (the *in vivo *state) and following organotypic and explant culture of the P1 and P7 cochleae were determined in the same manner.

To investigate neurite outgrowth from control and BDNF-treated P1 and P7 explants, single plane, confocal images of explants were captured using a 5× or 10× objective. NeuronJ (an ImageJ plugin) was used to trace and measure neurite lengths. SigmaPlot 11 (Systat Software Inc., Chicago, IL, USA) was used for all graphing and statistical analyses. The normal distribution of data was verified and statistical comparisons were made using one-way ANOVA's with *post hoc *Holm-Sidak tests and Students two-tailed, unpaired *t*-tests.

## Abbreviations

BDNF: brain-derived neurotrophic factor; IHC: inner hair cell; ISP: inner spiral plexus; NT-3: neurotrophin-3; OHC: outer hair cell; P: postnatal day; PBS: phosphate-buffered saline; SGN: spiral ganglion neuron.

## Competing interests

The authors declare that they have no competing interests.

## Authors' contributions

MB was involved in the design of the study, performed the experiments and data analysis and drafted the manuscript. AFR was involved in the conception and design of the study and contributed to the data analysis and manuscript. GH conceived the study, and participated in its design and coordination, data analysis and production of the manuscript. All authors read and approved the final manuscript.
